# A Fast Multiple Sampling Method for Low-Noise CMOS Image Sensors With Column-Parallel 12-bit SAR ADCs

**DOI:** 10.3390/s16010027

**Published:** 2015-12-26

**Authors:** Min-Kyu Kim, Seong-Kwan Hong, Oh-Kyong Kwon

**Affiliations:** Department of Electronics and Computer Engineering, Hanyang University, Seoul 133-791, Korea; gimmingyu@hanyang.ac.kr (M.-K.K.); seongkhong@hanyang.ac.kr (S.-K.H.)

**Keywords:** successive approximation register ADC, column parallel readout, CMOS image sensor

## Abstract

This paper presents a fast multiple sampling method for low-noise CMOS image sensor (CIS) applications with column-parallel successive approximation register analog-to-digital converters (SAR ADCs). The 12-bit SAR ADC using the proposed multiple sampling method decreases the A/D conversion time by repeatedly converting a pixel output to 4-bit after the first 12-bit A/D conversion, reducing noise of the CIS by one over the square root of the number of samplings. The area of the 12-bit SAR ADC is reduced by using a 10-bit capacitor digital-to-analog converter (DAC) with four scaled reference voltages. In addition, a simple up/down counter-based digital processing logic is proposed to perform complex calculations for multiple sampling and digital correlated double sampling. To verify the proposed multiple sampling method, a 256 × 128 pixel array CIS with 12-bit SAR ADCs was fabricated using 0.18 μm CMOS process. The measurement results shows that the proposed multiple sampling method reduces each A/D conversion time from 1.2 μs to 0.45 μs and random noise from 848.3 μV to 270.4 μV, achieving a dynamic range of 68.1 dB and an SNR of 39.2 dB.

## 1. Introduction

Recently, noise performance of CMOS image sensors (CISs) has become an important factor for images captured under low light conditions. The CIS typically uses a programmable gain amplifier (PGA) and a multiple sampling method to suppress noise caused by the pixel and readout circuit [1,2,3,4,5,6,7,8]. The PGA amplifies the pixel output and reduces noise with respect to the gain of the PGA. The readout circuit using the multiple sampling method repeatedly samples the pixel output and then averages the sampled pixel outputs to reduce noise by one over the square root of the number of samplings [7,8].

Several multiple sampling methods, such as the correlated multiple sampling (CMS), digital correlated multiple sampling (DCMS), and pseudo-multiple sampling (PMS) methods, have been studied for CISs with single-slope analog-to-digital converters (SS ADCs) [4,5,6]. The CMS method repeatedly integrates and averages the pixel output in the analog domain, but requires a power-consuming amplifier [4]. The DCMS method repeatedly converts the pixel output to a digital signal and averages the A/D conversion results in the digital domain. However, the total A/D conversion time increases in proportion to the number of samplings [5]. An alternative solution to DCMS, the PMS method which uses multiple ramp signals with different offsets is reported [6]. However, it requires an accurate ramp generator to control the offset of multiple ramp signals.

Several ADCs using the multiple sampling method have been researched to achieve low noise and overcome the drawbacks of SS ADC, which makes achieving short conversion time and high resolution difficult. A ΔΣ ADC easily achieves low noise by repeating sampling and integrating operations, but requires many clocks and a complex decimation filter [9]. An extended counting ADC (EC ADC) achieves short conversion time by sequentially converting the pixel output to the upper bit by using ΔΣ ADC and the lower bit by using cyclic ADC. However, an operational amplifier in EC ADC increases power consumption [10,11]. A successive approximation register ADC (SAR ADC) consumes less power due to its simple structure [3,12,13,14,15] and reduces noise by using the PMS method [14]. However, since the SAR ADC repeats the operation of the 1st A/D conversion, long A/D conversion time is required.

This paper proposes a fast multiple sampling (FMS) method for CIS with SAR ADC to achieve short conversion times and low noise. A 12-bit SAR ADC using the proposed FMS method repeatedly converts the pixel output to the lower 4-bit among the 12-bit output. Therefore, the required number of bit conversion steps is reduced to one-third. The 12-bit SAR ADC in the readout channel employs a 10-bit capacitor DAC with four scaled reference voltages to reduce the area. In addition, a simple digital processing logic consisting of a 3-input MUX and toggle flip-flop (T-F/F) is proposed to perform the complex calculations for multiple sampling and digital correlated double sampling (DCDS). In Section 2, the architecture of the developed CIS is described, along with the operating principle of the proposed FMS method. Section 3 presents the circuit implementations of the SAR ADC and the digital processing logic in the readout channel. In Section 4, the experimental results of the developed CIS are analyzed and compared with prior works. Finally, conclusions are given in Section 5.

## 2. CIS Architecture

### 2.1. Block Diagram

**Figure 1:**
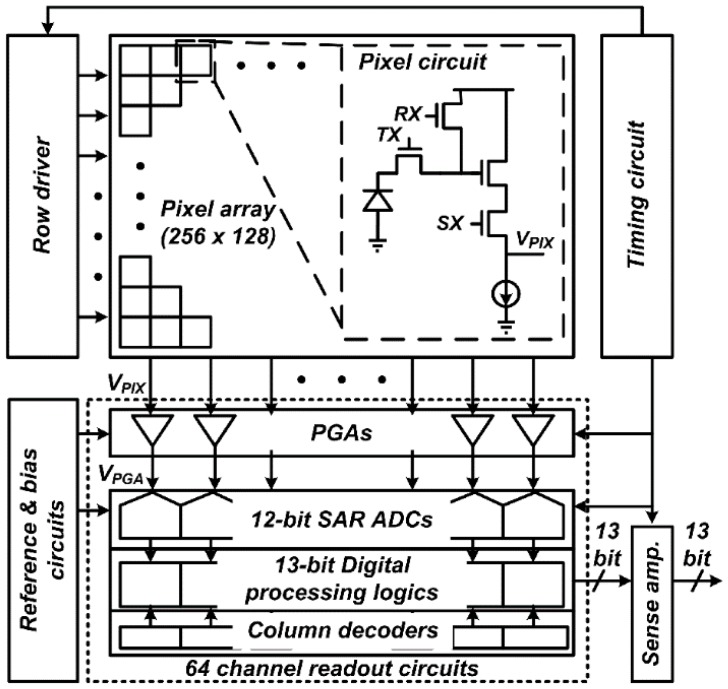
Block diagram of the developed CIS with a schematic of pixel circuit.

Figure 1 shows the block diagram of the developed CIS employing the proposed FMS method. The pixel array is composed of 256 × 128 pixels with a pixel size of 4.4 μm × 4.4 μm. Each readout channel, consisting of a PGA, a 12-bit SAR ADC, a digital processing logic, and a column decoder, has a pitch of 17.6 μm and converts the four column outputs of the pixel array to digital signals.

The PGA amplifies the pixel output, *V_PIX_*, and then the 12-bit SAR ADC using the proposed FMS method repeatedly converts the PGA output, *V_PGA_*, to a digital signal. The digital processing logic simultaneously performs the calculations for multiple sampling and DCDS. The output of the digital processing logic selected by the column decoder is transferred to the sense amplifier. To reduce the area and power consumption, four images obtained from each column output are externally combined to form an entire image. The timing circuit generates control signals for the row driver, readout circuit, and sense amplifier, while the bias and reference circuits provide the bias and reference voltages, respectively, for the PGA and 12-bit SAR ADC.

**Figure 2:**
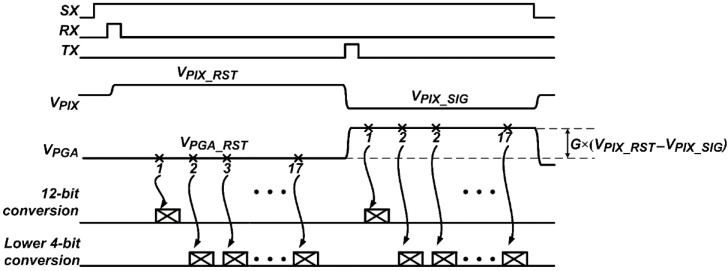
Operating sequence in a row line time.

Figure 2 shows the operating sequence of the developed CIS in a row line time. The pixels selected by *SX* sequentially generates a pixel reset voltage, *V_PIX_RST_*, and a photo-induced signal voltage, *V_PIX_SIG_*, according to the control signals, *RX* and *TX*, respectively. The PGA with a gain of *G* generates a PGA reset voltage, *V_PGA_RST_*, and an amplified pixel signal voltage, *V*_*PGA_*RST_ + *G* × (*V_PIX_RST_* – *V_PIX_SIG_*), according to the pixel output. The SAR ADC using the proposed FMS method has the maximum number of samplings of 17. At the first A/D conversion, the 12-bit SAR ADC converts *V_PGA_* to 12-bit, and then repeatedly converted *V_PGA_* to the lower 4-bit. The digital processing logic combines the sequential outputs of the comparator to obtain the A/D conversion result, and then subtracts the A/D conversion result of *V_PGA_RST_* from that of *V*_*PGA_*RST_ + *G* × (*V_PIX_RST_* – *V_PIX_SIG_*). Finally, the digital processing logic output becomes the A/D conversion result of *G* × (*V_PIX_RST_* – *V_PIX_SIG_*) [16,17].

### 2.2. Operating Principle of the Proposed FMS Method

**Figure 3:**
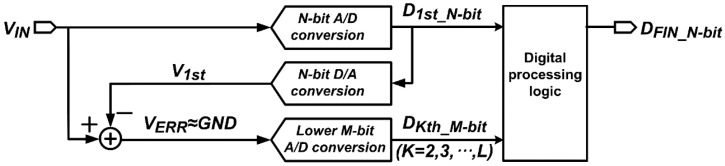
Signal flow diagram of the proposed FMS method.

Figure 3 shows the signal flow diagram of the proposed FMS method. The proposed FMS method operates in four steps: (1) An N-bit ADC converts the input voltage, *V_IN_*, to an N-bit digital signal, *D_1st_N-bit_*, for the first A/D conversion, where *V_IN_* is a constant for CIS applications; (2) An N-bit DAC converts *D_1st_N-bit_* to an analog voltage, *V_1st_*, and then the error voltage, *V_ERR_*, is obtained by subtracting *V_1st_* from *V_IN_*. With no quantization errors and noise, *V_1st_* is equal to *V_IN_*, and *V_ERR_* becomes *GND*. (3) An M-bit ADC converts *V_ERR_* to the lower M-bit among the N-bit output, where the range of *V_ERR_* is determined by the quantization error and noise. The proposed FMS method repeats the second and third steps (*L − 1*) times, where *L* is the number of samplings. Therefore, the lower M-bit conversion results, *D_Kth_M-bit_*’s, are repeatedly obtained from the second to Lth A/D conversion, where *D_Kth_M-bit_* corresponds to the Kth A/D conversion result. (4) To obtain the final A/D conversion result, *D_FIN_N-bit_*, the digital processing logic adds *D_1st_N-bit_* to the average value of *D_Kth_M-bit_*’s.

**Figure 4:**
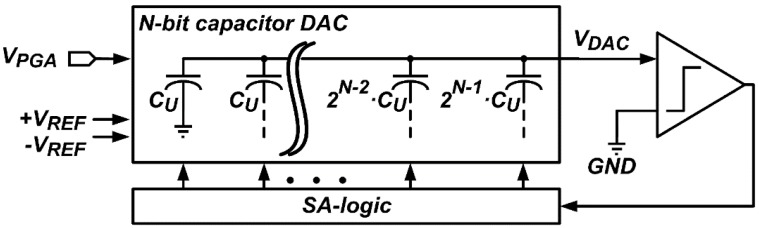
Block diagram of the N-bit SAR ADC.

Figure 4 shows the block diagram of the N-bit SAR ADC employing the proposed FMS method, without the use of an additional analog circuit for the N-bit D/A conversion in the second step and lower M-bit conversion in the third step. The N-bit SAR ADC consists of an N-bit capacitor DAC, a SAR logic, and a comparator. The reference voltages, +*V_REF_*, −*V_REF_*, and *GND* are used for the SAR ADC.

In the first step of the proposed FMS method, the N-bit capacitor DAC samples *V_IN_* by connecting the top and bottom plates of all capacitors to *V_IN_* and *GND*, respectively, and then its output, *V_DAC_*, became *V_IN_*. The comparator compares *V_DAC_* with *GND* to obtain the most significant bit (MSB), and the SAR logic connects the largest capacitor, *2^N−1^*·*C_U_*, to +*V_REF_* or −*V_REF_*. This operation is repeated until the least significant bit (LSB) is obtained. Then, the smallest capacitor, *C_U_*, is connected to +*V_REF_* or −*V_REF_*. After the first A/D conversion by the N-bit SAR ADC, *V_DAC_* is expressed as:

(1) $$V_{DAC}=V_{IN}+V_{1st\_NOISE}-\sum_{i=1}^{N}\left(D_{1st\_N-bit}[i]\times \frac{V_{REF}}{2^{i}}\right)\approx GND$$

where *D_1st_N-bit_*[*i*] corresponding to the *i*th bit of the first A/D conversion result has a value of ”1” or “−1”, and *V_1st_NOISE_* is an input-referred noise which includes the sampling and circuit noises at the first A/D conversion.

**Figure 5:**
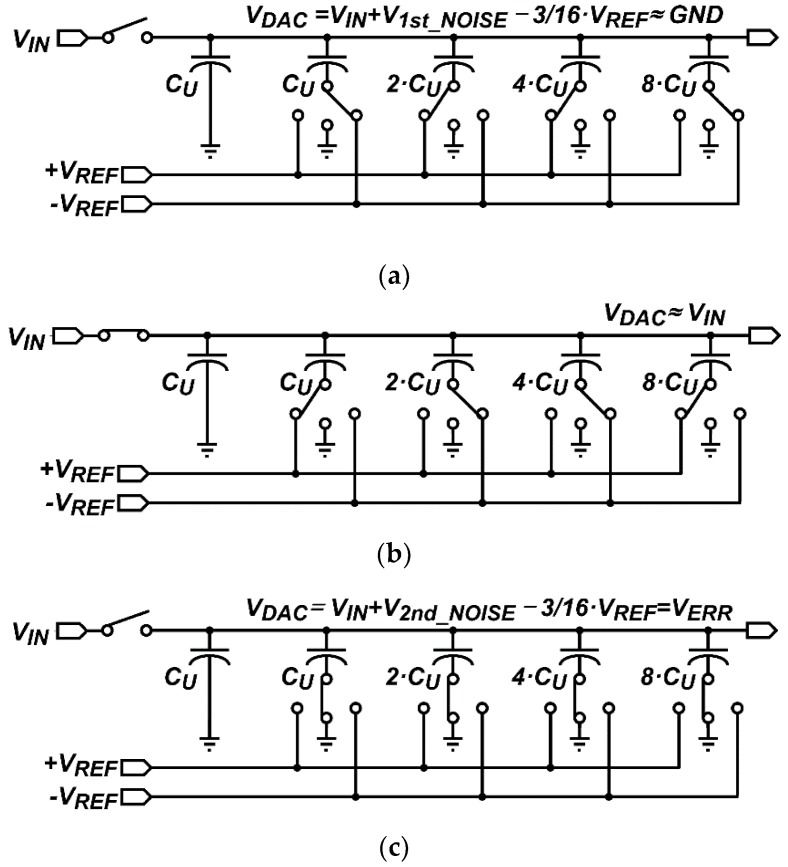
Schematics of the 4-bit capacitor DAC when the first A/D conversion result is “1001”: (**a**) when converting the 4-bit; (**b**) when sampling *V_IN_*, and (**c**) when obtaining *V_ERR_*.

Figure 5a shows an example of the 4-bit capacitor DAC when *D_1st_N-bit_* is ”1001”. The capacitors, 8·*C_U_*, 4·*C_U_*, 2·*C_U_*, and *C_U_*, are connected to −*V_REF_*, +*V_REF_*, +*V_REF_*, and −*V_REF_*, respectively, and *V_DAC_*, which is equal to *V_IN_* + *V_1st_NOISE_* − 3/16·*V_REF_*, converges to *GND*. In the second step, *V_IN_* is sampled again in the 4-bit capacitor DAC, and the capacitors, 8·*C_U_*, 4·*C_U_*, 2·*C_U_*, and *C_U_*, are connected to +*V_REF_*, −*V_REF_*, −*V_REF_*, and +*V_REF_*, respectively, as shown in Figure 5b, which are inversely connected as compared with Figure 5a. Afterwards, all capacitors are connected to *GND* as shown in Figure 5c, and *V_DAC_* becomes *V_IN_* + *V_2nd_NOISE_* − 3/16·*V_REF_* which is equal to *V_ERR_*, where *V_2nd_NOISE_* is an input-referred noise at the second A/D conversion. From the second to Lth A/D conversions, the N-bit capacitor DAC repeats the second step, and *V_DAC_* after the second step is expressed as:

(2) $$V_{DAC}=V_{IN}+V_{Kth\_NOISE}-\sum_{i=1}^{N}\left(D_{1st\_N-bit}[i]\times \frac{V_{REF}}{2^{i}}\right)=V_{Kth\_ERR}$$

where *V_Kth_NOISE_* and *V_Kth_ERR_* are the input-referred noise and error voltage at the Kth A/D conversion, respectively. Using Equations (1) and (2), *V_Kth_ERR_* can be simplified as:

(3) $$V_{Kth\_ERR}\approx GND-V_{1st\_NOISE}+V_{Kth\_NOISE}$$

*V_Kth_ERR_* has a Gaussian distribution around *GND − V_1st_NOISE_* and its minimum and maximum voltages are determined by *V_Kth_NOISE_*.

In the third step, since the range of *V_Kth_ERR_* is relatively small compared with that of *V_IN_*, *V_Kth_ERR_* can be converted to a digital signal using only lower capacitors in the N-bit capacitor DAC. After the N-bit SAR ADC converts *V_Kth_ERR_* to the lower M-bit among the N-bit output, *V_DAC_* is expressed as:

(4) $$V_{DAC}=V_{Kth\_ERR}-\sum_{i=1}^{M}\left(D_{Kth\_M-bit}[i]\times \frac{V_{REF}}{2^{N-M+i}}\right)\approx GND$$

where *D_Kth_M-bit_*[*i*] corresponding to the *i*th bit of the Kth A/D conversion result has a value of ”1” or “−1”. From the second to Lth A/D conversions, the SAR ADC repeats the second and third steps. After completing the total A/D conversion, the analog voltage corresponding to an average value of the lower M-bits, AVR[*V*(*D_Kth_M-bit_*)], is expressed as:

(5) $$\begin{array}{l} AVR\left[V\left(D_{Kth\_M-bit}\right)\right]=\frac{1}{L-1}\times \sum_{k=2}^{L}\sum_{i=1}^{M}\left(D_{Kth\_M-bit}[i]\times \frac{V_{REF}}{2^{N-M+i}}\right)\approx \frac{1}{L-1}\times \sum_{k=2}^{L}\left(V_{Kth\_ERR}-GND\right)\\ \approx \frac{1}{L-1}\times \sum_{k=2}^{L}\left(-V_{1st\_NOISE}+V_{Kth\_NOISE}\right)\approx -V_{1st\_NOISE}+\frac{1}{L-1}\times \sum_{k=2}^{L}V_{Kth\_NOISE} \end{array}$$

where (*L − 1*) is the repeated number of the second and third steps at the number of samplings of *L*. The final A/D conversion result, *D_FIN_N-bit_*, is obtained by adding *D_1st_N-bit_* and the average value of the lower M-bits, while the analog voltage corresponding to *D_FIN_N-bit_*, *V*(*D_FIN_N-bit_*), can be expressed as:

(6) $$\begin{array}{l} V\left(D_{FIN\_N-bit}\right)=\sum_{i=1}^{N}\left(D_{FIN\_N-bit}[i]\times \frac{V_{REF}}{2^{i}}\right)\\ =\sum_{i=1}^{N}\left(D_{1st\_N-bit}[i]\times \frac{V_{REF}}{2^{i}}\right)+\frac{1}{L-1}\times \sum_{k=2}^{L}\sum_{i=1}^{M}\left(D_{Kth\_M-bit}[i]\times \frac{V_{REF}}{2^{N-M+i}}\right)\\ \approx V_{IN}-GND+\frac{1}{L-1}\times \sum_{k=2}^{L}V_{Kth\_NOISE} \end{array}$$

where *D_FIN_N-bit_*[*i*] corresponding to the *i*th bit of the final A/D conversion result has a value of ”1” or “−1”. Since the effect of *V_Kth_NOISE_* on *V*(*D_FIN_N-bit_*) decreases by averaging *V_Kth_NOISE_*, *V*(*D_FIN_N-bit_*) converges to *V_IN_ − GND* as *L* increases.

## 3. Circuit Implementation

### 3.1. Design of 12-bit SAR ADC Using the Proposed FMS Method

**Figure 6:**
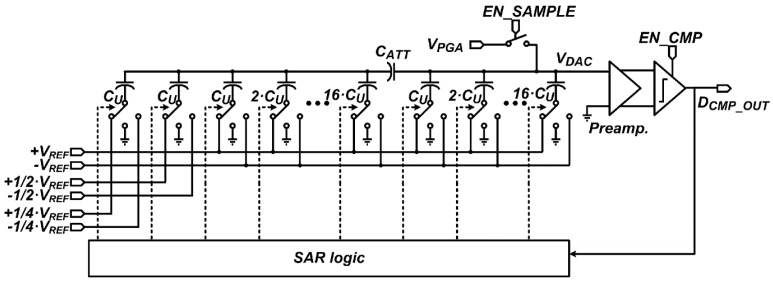
Schematic of the 12-bit SAR ADC.

Figure 6 shows a schematic of the 12-bit SAR ADC which converts the PGA output, *V_PGA_*, to a digital signal. The PGA in [2,3], which has gains of ×1, ×2, and ×4, is used for the developed CIS. To reduce the area, the SAR ADC uses a 10-bit capacitor DAC, which has a split capacitor structure with an attenuation capacitor, *C_ATT_*, instead of a 12-bit capacitor DAC. To obtain the additional lower 2-bit, four scaled reference voltages, +1/2·*V_REF_*, +1/4·*V_REF_*, −1/4·*V_REF_*, and −1/2·*V_REF_*, are used in the 10-bit capacitor DAC. The reference voltages, +*V_REF_*, −*V_REF_*, and *GND*, are generated via an off-chip DAC, whereas the scaled reference voltages are generated by using an internal R-string. All reference voltages are provided to the 12-bit SAR ADC via on-chip reference buffers. At the rising edge of the control signal, *EN_CMP*, the clocked comparator compares two outputs of the preamplifier which amplifies the difference between *V_DAC_* and *GND*. The SAR ADC converts *V_PGA_* to 12-bit at the first conversion, and then repeatedly converts *V_PGA_* to the lower 4-bit. 12 latches and four latches operate as SAR logic at the 12-bit and lower 4-bit conversions, respectively. The SAR logic sequentially stores the output of the clocked comparator and selects the reference voltages connected to the capacitors in the 10-bit capacitor DAC.

**Figure 7:**
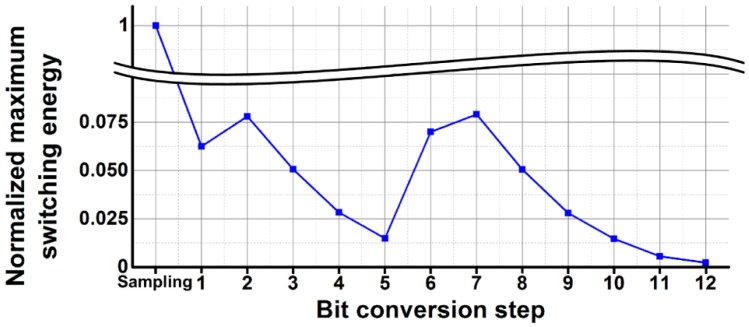
Normalized maximum switching energies of the capacitor DAC.

Figure 7 shows the maximum switching energies of the capacitor DAC according to the bit conversion step of the 12-bit conversion. Since charges in the capacitor DAC are provided from the reference voltages, the switching energy determines the settling time for each bit conversion step [12], and it is calculated using equations in [15]. When the capacitor DAC is reset and samples *V_PGA_*, all the capacitors are simultaneously charged to *V_PGA_* and the largest switching energy is required in the bit conversion steps. The maximum switching energy decreases with an increase of the bit conversion step with an exception of the second bit, sixth bit, and seventh bit conversion steps, due to the split capacitor structure. Considering the driving capability of the PGA and the decrease in switching energy, the 12-bit SAR ADC is designed to take 200 ns to sample *V_PGA_*, 100 ns to convert each upper 8-bit, 50 ns to convert each lower 4-bit, and 100 ns to obtain *V_Kth_ERR_*. Therefore, each A/D conversion time becomes 1.2 μs for the first conversion, and then is reduced to 0.45 μs for the second to seventeenth conversion.

**Figure 8:**
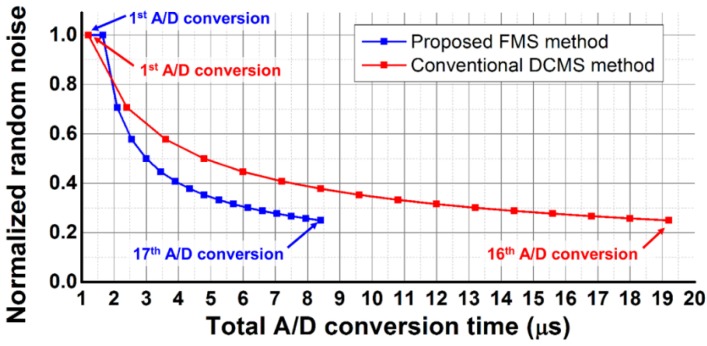
Theoretical normalized noise of the SAR ADC output.

Figure 8 shows the theoretical normalized noise of the SAR ADC with the proposed FMS method and conventional DCMS method, which repeats the same operations for each A/D conversion, according to the total A/D conversion time. When the number of samplings is *L*, the noise for the proposed FMS method decreases by one over the square root of (*L − 1*), according to Equation (6), and the noise for the conventional DCMS method decreases by one over the square root of *L*. However, when the number of samplings is 17, which is the maximum number for the developed CIS, the total A/D conversion time of the SAR ADC using the proposed FMS method is reduced to 8.4 μs from 19.2 μs, which is that of the SAR ADC using the conventional DCMS method.

### 3.2. Design of Digital Processing Logic

**Figure 9:**
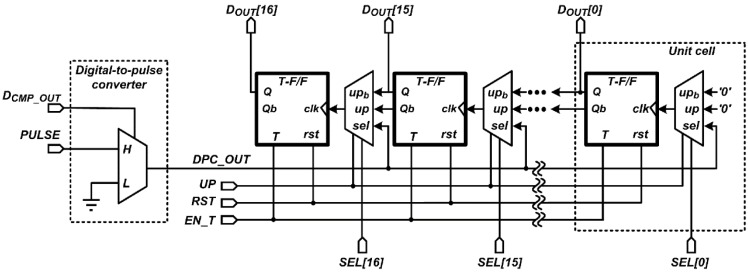
Block diagram of the digital processing logic.

Figure 9 shows the block diagram of the proposed up/down counter-based digital processing logic for the 12-bit SAR ADC employing the proposed FMS method. The proposed digital processing logic consists of a digital-to-pulse converter (DPC) and 17 unit cells for the maximum number of samplings of 17, of which each unit cell consists of a T-F/F and 3-input MUX. The T-F/F output or its inverting output, *Q* or *Qb*, respectively, is transferred to the next T-F/F though the 3-input MUX according to the control signal, *UP*. The DPC, which consists of a 2-input MUX, selects a signal, *GND* or *PULSE*, according to the comparator output, *CMP_OUT*, and its output, *DPC_OUT*, is applied to one of the T-F/Fs through the 3-input MUX selected by the control signal, *SEL*. The control signal, *EN_T*, becomes low to maintain the output of the T-F/F when the control signal, *UP* or *SEL*, changes. The digital processing logic subtracts the A/D conversion result of the PGA reset voltage from that of the amplified pixel signal voltage, and the developed CIS uses the upper 13-bit, from *D_OUT_*[*16*] to *D_OUT_*[*5*], among the 17-bit outputs of the digital processing logic for displaying the captured image. The MSB generated via subtraction is used as a sign bit.

**Figure 10:**
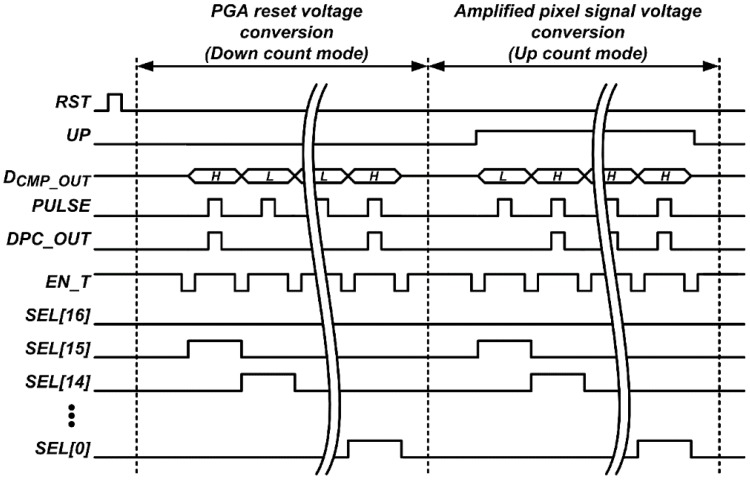
Timing diagram of the digital processing logic.

Figure 10 shows the timing diagram of the digital processing logic. Before converting the PGA reset voltage, all T-F/Fs are reset by the control signal, *RST*, and the digital processing logic acts as a down counter by setting the control signal, *UP*, to low. At the first A/D conversion, the 12-bit SAR ADC converts the PGA reset voltage to 12-bit, and *D_CMP_OUT_* from the MSB to the LSB is sequentially provided to the DPC. When *D_CMP_OUT_* is low or high, *DPC_OUT* keeps or toggles the output of the T-F/F, respectively, starting from *D_OUT_*[15] to *D_OUT_*[4] sequentially. Therefore, *D_OUT_* becomes two’s complement of the first A/D conversion result. From the second to seventeenth A/D conversion, the 12-bit SAR ADC converts the PGA reset voltage to the lower 4-bit, and *DPC_OUT* is sequentially provided from the fourteenth to seventeenth T-F/F, which corresponds to the lower 4-bit. Therefore, the lower 4-bit conversion results are repeatedly subtracted from *D_OUT_* 16 times, and then averaged by 16. Therefore, the upper 13-bit, from *D_OUT_*[16] to *D_OUT_*[4], becomes a final A/D conversion result of the PGA reset voltage. Afterwards, the 12-bit SAR ADC converts the amplified pixel signal voltage to 12-bit. The same operations as those in the PGA reset voltage conversion are repeated, with the exception of the digital processing logic, which acts as an up counter by setting *UP* to high. Therefore, the upper 13-bit becomes the difference between the A/D conversion results of the PGA reset voltage and the amplified pixel signal voltage.

The proposed up/down counter-based digital processing logic uses a T-F/F and 3-input MUX per unit cell. As a result, the number of transistors per unit cell is reduced by 29% compared with that of the SAR ADC described in [3]. Moreover, the proposed digital processing logic is also applicable to the multi-bit cyclic ADC with the error correction algorithm in [18] by controlling the number of output pulses from the DPC without the use of additional circuits in the unit cell. The multi-bit cyclic ADC sequentially generates the multi-bit per clock cycle to compensate for the comparator offset.

## 4. Experimental Results

**Figure 11:**
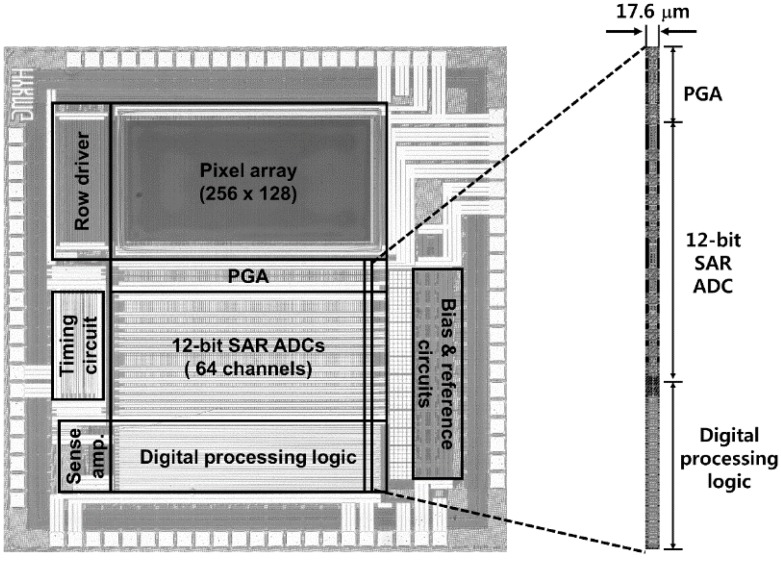
Photomicrograph and readout channel layout of the developed CIS.

The proposed FMS method is verified using a 256 × 128 pixel array CIS with column-parallel 12-bit SAR ADCs. Figure 11 shows the chip photomicrograph and readout channel layout of the developed CIS which is fabricated using a 0.18 μm 1-poly 4-metal CMOS process. The developed CIS occupies an area of 2.35 mm × 2.35 mm.

The developed CIS uses supply voltages of 2.8 V for the pixel array, analog circuit, and 1.8 V for digital circuit. The total power consumption excluding PAD power is obtained from post layout simulation results under the operating conditions at the number of sampling of 17 and 90 frames/s. It has 4.4 mW which includes the power consumptions of the pixel array of 0.79 mW, reference and bias circuits of 1.14 mW, and readout channel array of 2.3 mW. In a readout channel, the PGA, SAR ADC, and digital processing logic consume 20.6 μW, 11.3 μW, and 4.3 μW, respectively.

**Figure 12:**
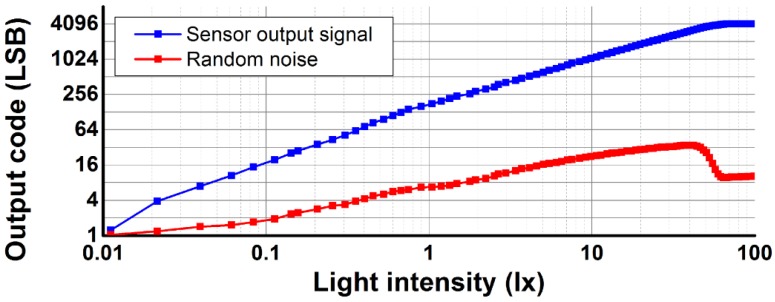
Measured output signal and random noise according to light intensity.

Figure 12 shows the measured sensor output signal and random noise of the developed CIS according to light intensity, which is obtained from the average and standard deviation values of 100 images, respectively, at a PGA gain of ×1 and the number of samplings of 17. The output signal and random noise increases linearly according to the light intensity. However, the nonlinearity of the conversion capacitor and source follower in the pixel causes the non-linear behavior [3,19]. The light sensitivity obtained from the slope of the output signal is 6.2 V/lx·s. Random noise has a minimum value of 1.2 LSB (270.4 μV) in near-dark condition, where flickers and thermal noises of the pixel and readout circuits are the dominant sources of noise. As the light intensity increases, random noise increases due to photon shot noise. At a light intensity of 40 lx, the measured output signal and random noise have 3110 LSB and a maximum value of 34 LSB, respectively. An output signal of 3110 LSB corresponds to the full well capacity of 11.4 ke^−^ with a conversion gain of 60 μV/e^−^ [19]. The dynamic range obtained from the ratio of the full well capacity to the minimum random noise measured in near-dark conditions is 68.1 dB. The signal-to-noise ratio (SNR) obtained from the ratio of the output signal to random noise is 39.2 dB at a light intensity of 40 lx.

**Figure 13:**
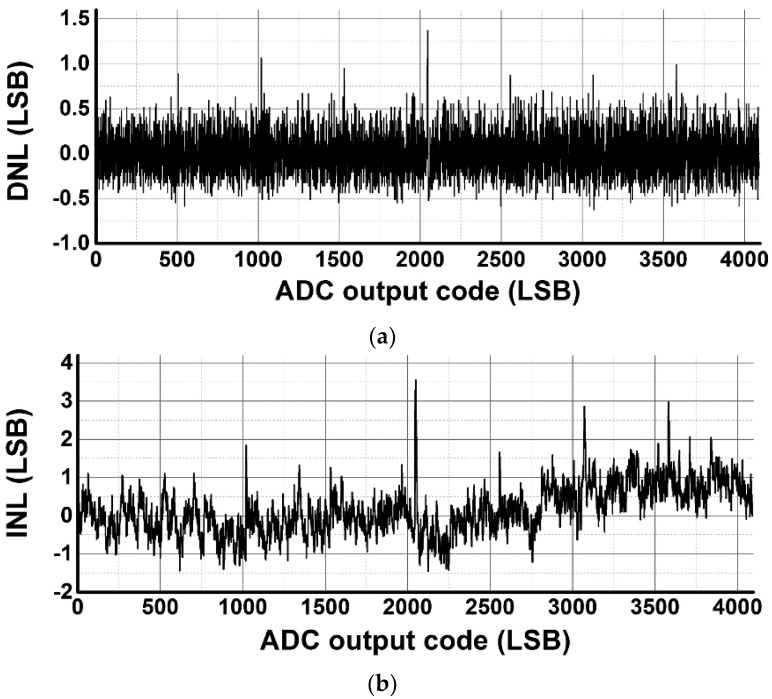
Measured (**a**) DNL and (**b**) INL of the SAR ADC.

Figure 13 shows the measured differential non-linearity (DNL) and integral non-linearity (INL) of the 12-bit SAR ADC for the developed CIS. The measurement results of the DNL and INL are −0.62/+1.37 LSB and −1.42/+3.55 LSB, respectively. Since code saturations are caused by the parasitic capacitors connected to upper capacitors in the capacitor DAC, the peaks of the DNL and INL repeatedly occur. In addition, a mismatch of *C_ATT_* repeatedly causes peaks of DNL and INL in each 128 LSB over the full code range. Linearity of the CIS is not affected by the INL of the ADC, but are determined by the photon shot noise and photo conversion nonlinearity [3]. In addition, the error of *V_ERR_* is not occurred due to capacitance mismatch of the capacitor DAC since the D/A conversion error of the capacitor DAC is cancelled out while converting the input voltage to *D_1st_Nbit_* and generating *V_ERR_* from *D_1st_N-bit_*. However, since the asymmetry of *+V_REF_* and *−V_REF_* causes an offset error in *V_ERR_*, the A/D conversion range for the lower 4-bit conversion should be wider than the offset error.

**Figure 14:**
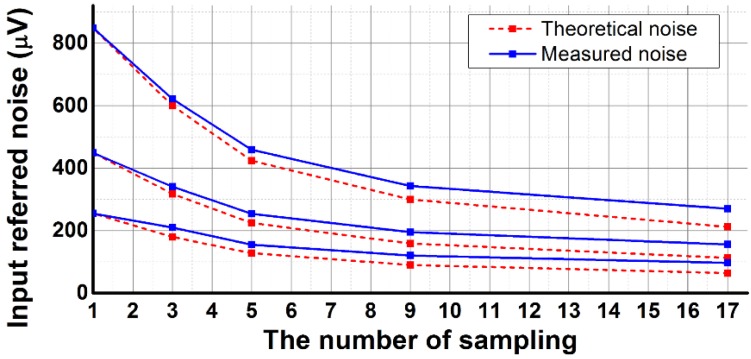
Measured and theoretical input referred noise.

Figure 14 shows the measured and theoretical input referred noises according to the number of samplings and PGA gain at the same exposure time. As the number of samplings increased from 1 to 17, the input referred noise decreases from 848.3 μV to 270.4 μV, 449.2 μV to 155.8 μV, and 255 μV to 96.5 μV, for a PGA gain of ×1, ×2, and ×4, respectively. *V_Kth_ERR_*, which is the sum of input referred noises at the 1st and Kth conversions, is repeatedly converted to lower 4-bit after 1st A/D conversion.

Therefore, lower 4-bit for the fast conversion is derived by considering a measured input referred noise of 848.3 μV, which corresponds to 3.9 LSB, at the number of samplings of 1 and a PGA gain of ×1. Theoretically, for the number of samplings of *L*, the input referred noise should be decreased by one over the square root of (*L − 1*), but the measured input referred noise is greater than the theoretical noise due to the linearity error of the lower 4-bit conversion of the SAR ADC. The lower 4-bit conversion results after the first conversion exhibits a Gaussian distribution due to temporal noise, but linearity error distorts the distribution. In addition, the measured input referred noise proportionally decreases according to the PGA gain because, for the input referred noise, the noise caused by the readout circuit is dominant compared with what is caused by the pixel circuit [1]. The developed CIS achieves the lowest input referred noise of 96.5 μV at the number of samplings of 17 and a PGA gain of ×4.

**Figure 15:**
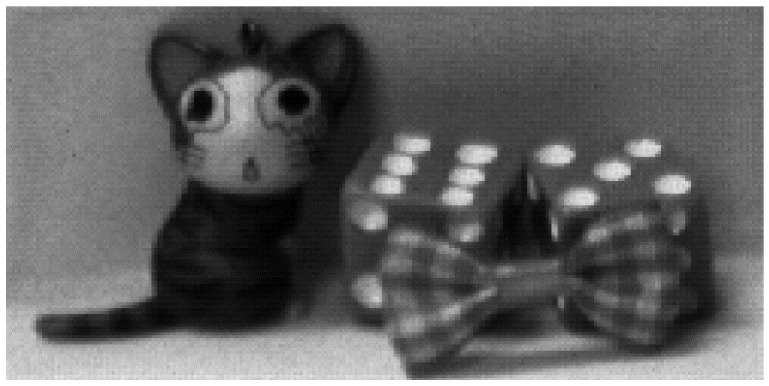
Captured 12-bit image at the number of samplings of 17.

**Figure 16:**
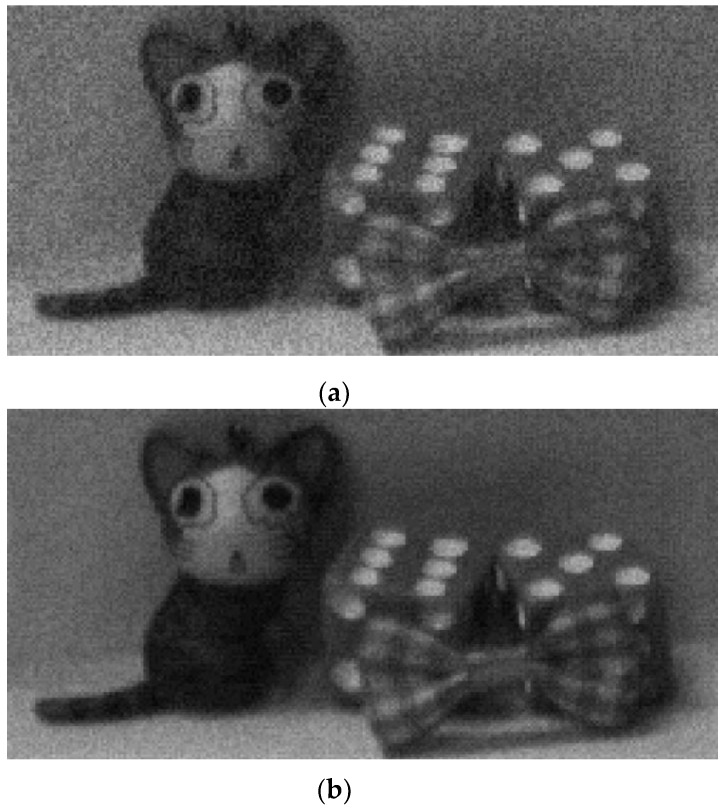
Captured lower 5-bit images in short exposure time for the following number of samplings: (**a**) 1 and (**b**) 17.

Figure 15 shows the captured image of the developed CIS at the number of samplings of 17 and a PGA gain of ×1. The captured image exhibits a 12-bit resolution, but it is difficult to evaluate the noise performance of the CIS with the naked eye because the monitor system generally features an 8-bit resolution. To solve the above problem, images are captured in short exposure time and are displayed by using the lower 5-bits among the 12-bit CIS outputs, corresponding to a digital gain of ×128. Figure 16a,b shows the captured images for the following number of samplings, 1 and 17, respectively, at a PGA gain of ×1, in which Figure 16b has less noise than Figure 16a.

The performance of the developed CIS is summarized in Table 1 and compared with prior CISs using column-parallel SAR ADCs in Table 2. The figure of merit, representing noise and energy efficiency, was defined as:

(7) $$FOM=\frac{Power\times Noise}{Pixel\_rate}$$

where *Pixel_rate* is a product of the total number of pixels and the frame rate. The CIS employing the proposed FMS method achieves the best FOM of 145 μV·nJ by simultaneously reducing the total A/D conversion time and random noise.

**Table 1:** Performance summary.

Parameter	Value
Process	0.18 μm 1-poly 4-metal CMOS process
Supply voltage	2.8 V/1.8 V
Chip size	2.35 mm × 2.35 mm
Pixel array size	256 (H) × 128 (V)
Maximum frame rate	90 frames/s
Pixel size	4.4 μm × 4.4 μm
Conversion gain	60 μV/e^−^
Full well capacity	11.4 ke^−^
Sensitivity	6.2 V/lx·s
Column FPN at dark	0.17 LSB
SNR	39.2 dB
Dynamic range	68.1 dB
ADC input range	0.9 V
ADC resolution	12-bit
DNL	−0.62/+1.37 LSB
INL	−1.42/+3.55 LSB
Power consumption	4.4 mW

**Table 2:** Comparison with prior CISs with column-parallel SAR ADCs.

Parameter	This Work	[3]	[12]	[13]	[14]	[20]	[21]
Pixel array size	256 × 128	4112 × 2186	1280 × 800	920 × 256	644 × 488	54 × 50	64 × 45
Frame rate (frame/s)	90	60	35	9	120	7.4	21.2
ADC Resolution (bit)	12	14	11	9	14	10	8
Random noise (μV_rms_)	96.5 (0.44 LSB)	130.5	1500	5300	83	0.98 LSB	0.5 LSB
Power consumption (mW)	4.4	108.5	40	1.1	78	0.014	0.021
FOM (μV·nJ)	145	265	1674	28147	171	-	-

## 5. Conclusions

In this paper, a fast multiple sampling method for CISs with column-parallel 12-bit SAR ADCs is proposed. The SAR ADC repeatedly converts a pixel output to 4-bit after the first 12-bit A/D conversion. As a result, each A/D conversion time after the first A/D conversion is reduced to 37.5% of the first A/D conversion time, and the total A/D conversion time at the number of samplings of 17 is reduced to 44% of that of the SAR ADC using conventional DCMS method. The 12-bit SAR ADC uses a 10-bit capacitor DAC with an attenuation capacitor and four scaled reference voltages to reduce the area. A simple up/down counter-based digital processing logic, consisting of a 3-input MUX and T-F/F is proposed to perform complex calculations for multiple sampling and DCDS. The measurement results shows that random noise decreases from 848.3 μV to 270.4 μV by using the proposed multiple sampling method, and the best FOM of 145 μV·nJ is achieved. Therefore, the proposed multiple sampling method is suitable for low-noise, high-frame rate CISs with column-parallel SAR ADCs.
